# A nucleoside-modified mRNA vaccine prevents enterovirus A71 infection in mouse model

**DOI:** 10.3389/fimmu.2025.1535758

**Published:** 2025-02-12

**Authors:** Fengyu Chi, Xu Zhang, Dong Zhang, Airu Zhu, Zhen Zhuang, Zhaoyong Zhang, Zhenjie Zhang, Chuansong Quan, Kaixiao Nie, Juan Li, Chunhong Yin, Jie Tong, Yuming Li

**Affiliations:** ^1^ School of Public Health, Shandong First Medical University and Shandong Academy of Medical Sciences, Ji’nan, China; ^2^ Key Laboratory of Emerging Infectious Diseases in Universities of Shandong, Shandong First Medical University and Shandong Academy of Medical Sciences, Ji’nan, China; ^3^ State Key Laboratory of Respiratory Disease, National Clinical Research Centre for Respiratory Disease, National Centre for Respiratory Medicine, Guangzhou Institute of Respiratory Health, The First Affiliated Hospital of Guangzhou Medical University, Guangzhou, Guangdong, China; ^4^ Infectious Disease Control Institute, Shandong Center for Disease Control and Prevention, Ji’nan, China; ^5^ College of Life Science, Institute of Life Science and Green Development, Hebei University, Baoding, China; ^6^ Shandong Provincial Qianfoshan Hospital, The First Affiliated Hospital of Shandong First Medical University, Ji’nan, China

**Keywords:** human enterovirus A71, non-enveloped virus, VP1, mRNA vaccine, immune response

## Abstract

**Introduction:**

Human Enterovirus A71 (EV-A71) is the primary pathogen responsible for severe hand, foot, and mouth disease (HFMD). Vaccination plays a crucial role in controlling its spread. Although inactivated vaccines have been approved, there is growing interest in developing new candidates using advanced platforms. mRNA vaccines, widely used for enveloped viruses, are less studied for non-enveloped viruses like EV-A71. This study investigates the potential of an mRNA vaccine targeting the EV-A71 VP1 protein.

**Methods:**

A nucleoside-modified mRNA vaccine encoding the VP1 protein of EV-A71, encapsulated in lipid nanoparticles (LNPs), was developed. Immunogenicity and protective efficacy were evaluated in BALB/c and neonatal A129 mice, respectively. Immune responses were assessed by ELISA, micro-neutralization assays, ELISpot, and intracellular cytokine staining (ICS). Passive protection was tested by transferring immune sera to neonatal mice challenged with EV-A71.

**Results:**

The VP1 mRNA-LNP vaccine elicited robust humoral and cellular immunity, including high levels of VP1-specific IgG, neutralizing antibodies, and a Th1-biased T-cell response. Notably, the mRNA vaccine outperformed the inactivated vaccine in eliciting cellular immunity. Immune sera provided complete protection against lethal EV-A71 challenge, significantly reducing viral load and pathology.

**Discussion:**

This study demonstrates that the mRNA vaccine exhibits significant potential for combating non-enveloped viruses. These findings highlight the promising role of mRNA platforms in advancing vaccine development against non-enveloped viral pathogens, offering new avenues for future research and clinical applications.

## Introduction

Enteroviruses (EVs) are classified as members of the *Enterovirus* genus within the *Picornaviridae* family. These non-enveloped, single-stranded RNA viruses have genomes encapsulated in viral capsids, forming symmetrical icosahedral particles approximately 20-30 nm in diameter (1, 2). The *Enterovirus* genus includes 12 *Enterovirus* species (A-L) and 3 *Rhinovirus* species (RV A-C). Enterovirus A71 (EV-A71) belonging to the enterovirus A species is transmitted via the fecal-oral route (2, 3). EV-A71 was first isolated from fecal specimens of an infant with aseptic meningitis in California, USA, in 1969 (4). Since then, numerous outbreaks and epidemics of EV-A71 have been reported worldwide (5–8), with notable occurrences in the Asia-Pacific region since the late 1990s (9). EV-A71 predominantly affects children under five years old and is one of the main causative agents of hand, foot, and mouth disease (HFMD), which typically resolves within 1–2 weeks as a self-limiting illness. However, in severe cases, EV-A71 can cause neurological complications, leading to a poor prognosis or even death, posing a significant health threat to infants and young children. Therefore, EV-A71 is recognized as the most significant neurotropic enterovirus after poliovirus (10–12).

The EV-A71 genome is approximately 7,500 nucleotides long and encodes four structural proteins (VP1 to VP4) and seven non-structural proteins (2A to 2C and 3A to 3D). Structural proteins VP1 to VP4 firstly combine to form a protomer, with sixty protomers assemble into a viral capsid that encapsulates the viral genome (13). Of these proteins, VP1, VP2, and VP3 are exposed on the surface of the capsid, while VP4 resides internally (13, 14). VP1 is the most immunodominant structural protein consisting of 297 amino acids and contains major neutralizing epitopes. It plays a crucial role in virus adsorption, penetration, and uncoating during the EV-A71 lifecycle, making it a primary target for molecular research and vaccine development (15–17).

Currently, no specific drugs are approved for EV-A71, so supportive therapy is the primary treatment for EV-A71-related diseases. Vaccination is the most effective and cost-efficient strategy for EV-A71 prevention. Recent research on EV-A71 vaccines has mainly focused on inactivated vaccines (18, 19), virus-like particles (VLP) (20–22), live attenuated vaccines (23, 24), and subunit vaccines (25, 26). Among these, only inactivated EV-A71 vaccine has completed human clinical trials, while the other candidates are still in preclinical animal evaluation (27). Between 2015 and 2017, the China Food and Drug Administration (CFDA) approved the commercialization of three inactivated vaccines targeting the EV-A71 C4 sub-genotype (28–30). Phase III clinical trials demonstrated that all three vaccines effectively reduced EV-A71-related HFMD (27). However, inactivated vaccines face challenges, including high production costs, long development timelines, and potentially weakened immunogenicity, which may result in inadequate stimulation of cellular immune responses (22). Moreover, increasing evidence suggests that intra- and intertypic recombination, alongside mutations in co-circulating EV-A71 strains, has driven rapid viral evolution, posing potential challenges for inactivated vaccines (31, 32).

As a promising and versatile vaccine platform, mRNA-based vaccines are applicable to both infectious diseases and cancer. They offer several advantages, including shorter development cycles, strong immunogenicity, favorable safety profiles, and adaptability to mutations (33, 34). Recent breakthroughs in RNA molecular modification and delivery systems, coupled with the urgent need for rapid development and large-scale production of vaccines and antiviral drugs for emerging infectious diseases, have greatly accelerated mRNA vaccines research (35). In December 2020, the first two SARS-CoV-2 mRNA vaccines, developed by Moderna and Pfizer/BioNTech, received Emergency Use Authorization (EUA) in the United States, demonstrating over 90% efficacy in Phase III clinical trials (36, 37). Since then, additional SARS-CoV-2 mRNA vaccines received regulatory approval. Recently, the first non-COVID-19 mRNA vaccine for Respiratory Syncytial Virus (RSV) from Moderna was approved by the U.S. Food and Drug Administration (FDA) (38). Currently, several mRNA vaccine candidates targeting infectious agents such as the influenza virus, Zika virus, Nipah virus, and cytomegalovirus, are in different stages of clinical trials (39). Additionally, mRNA vaccines targeting non-communicable diseases such as melanoma, lymphoma, and colorectal cancer are also under various phases of development (40).

In this study, we developed an mRNA vaccine candidate targeting EV-A71, utilizing the VP1 structural protein coding sequence encapsulated within lipid nanoparticles (LNPs). Immunization experiments in mice demonstrated that the vaccine effectively elicits robust humoral and cellular immune responses, successfully protecting newborn mice from infection.

## Results

### EV-A71 VP1 mRNA-LNP production and *in vitro* characterization

**Figure 1:**
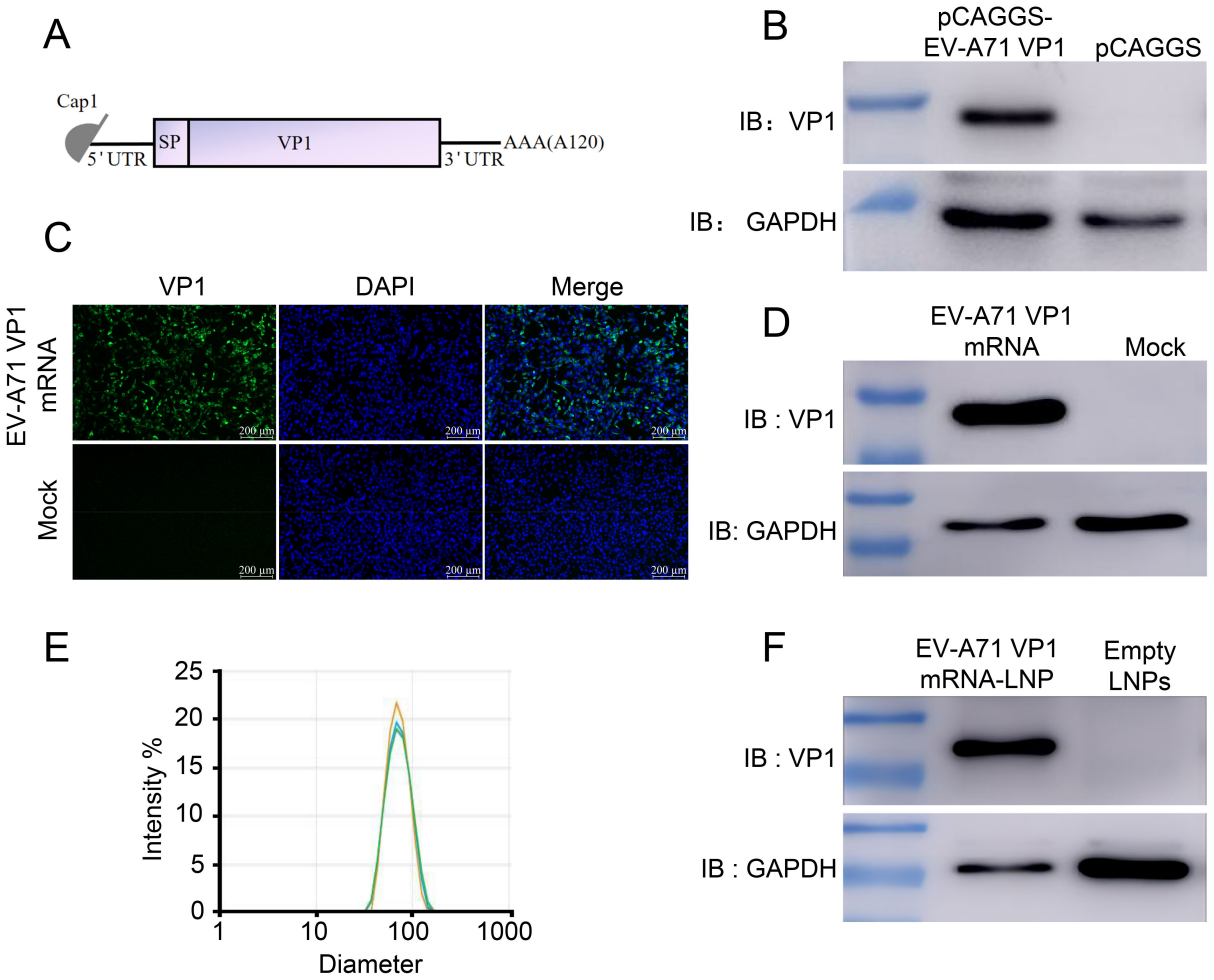
Design and characterization of mRNA vaccine encoding the EV-A71 VP1. **(A)** Schematic representation of the mRNA vaccine design. The mRNA consists of 5’ cap followed by a 5’UTR, a signal peptide, full-length VP1, a 3’UTR, and a poly (A) tail. **(B)** pCAGGS-EV-A71 VP1 was transfected into HEK 293T cells, and VP1 expression was identified using Western blot (WB). VP1 expression following mRNA-VP1 transfection into HEK 293T cells was assessed using immunofluorescence assay (IFA) **(C)** and WB **(D)**. **(E)** The size distribution of VP1-LNPs was measured using dynamic light scattering (DLS). **(F)** VP1-LNPs were transfected into RD cells, and VP1 expression was confirmed using WB.

Previous studies have demonstrated that VP1 serves as the receptor-binding protein for EV-A71 infection in cells and is the key target for vaccine development (16). Therefore, we designed a mRNA vaccine encoding the full-length of VP1 from the EV-A71 JN315 strain (C4 sub-genotype) (**Figure 1A**; **Supplementary Table S1**). First, codon-optimized VP1 sequence was cloned into a eukaryotic expression vector (pCAGGS) and the expression of VP1 was evaluated post transfection into HEK 293T cells. The results exhibited the successful expression of VP1, suggesting the efficient usage of VP1 mRNA codon (**Figure 1B**). Next, we conducted *in vitro* transcription to synthesize mRNA with N1-methyl-pseudouridine nucleoside substituting of uridine. Subsequently, this mRNA was transfected into RD cells and its expression was validated using immunofluorescence (**Figure 1C**) and Western blot (**Figure 1D**). Following this, the mRNA was encapsulated in lipid nanoparticles (LNP) forming into mRNA nanoparticle vaccines with approximately 84 nm in size (**Figure 1E**). Finally, VP1 mRNA-LNP was transfected into A549 cells and its expression *in vitro* was confirmed as well (**Figure 1F**). All these results demonstrate that the VP1 mRNA-LNP vaccine we developed can be successfully expressed *in vitro* and could be used for further analysis *in vivo*.

### EV-A71 VP1 mRNA-LNP immunization elicited robust humoral immune responses in BALB/c mice

**Figure 2:**
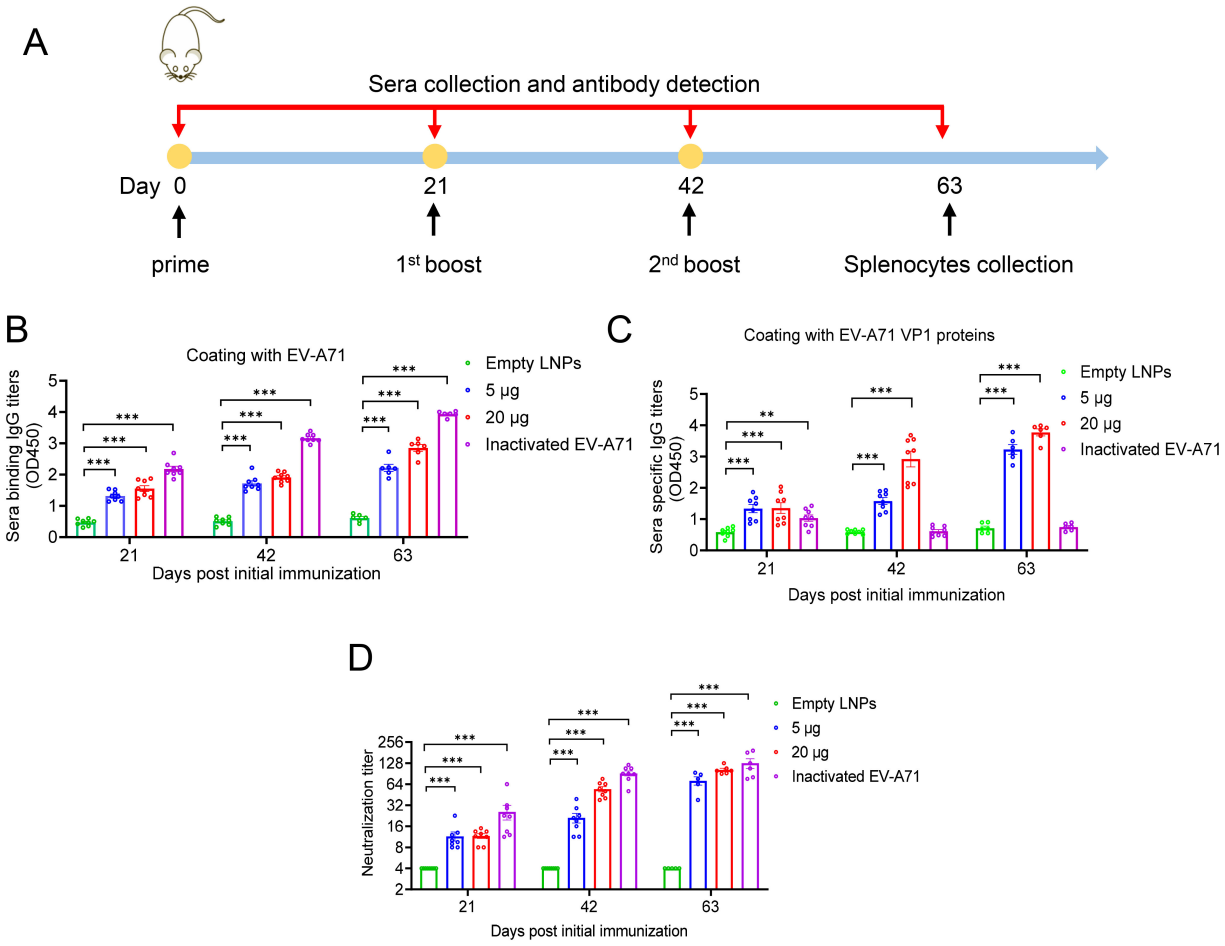
Humoral immune response induced by EV-A71 VP1 mRNA vaccines and inactivated EV-A71 vaccines in mice. **(A)** Timeline of mice immunization and sample collection. Specific IgG antibody titers in mouse sera were measured using ELISA plates coated with VP1 protein **(B)** and EV-A71 virus particles **(C)**, respectively. **(D)** Neutralizing antibody titers in mouse sera was measured by micro-neutralization assay. Data are shown as the mean ± SEM. Statistical significance was determined using a two-way ANOVA with multiple comparison tests. **p < 0.01, ***p < 0.001.

To evaluated the immunogenicity of our mRNA-based EV-A71 vaccine, groups of BALB/c mice were immunized intramuscularly (i.m.) with either a high dose (20 µg) or a low dose (5 µg) of VP1 mRNA-LNP three times at 3-week intervals. Empty LNPs served as the negative control, while inactivated EV-A71 vaccine was used as the positive control (**Figure 2A**). Mice sera were collected at days 21, 42, and 63 post initial vaccination and analyzed by ELISA. The results indicated that EV-A71 mRNA vaccine induced a strong production of EV-A71-specific IgG antibodies. Both the 5 µg and 20 µg doses of VP1 mRNA-LNP generated high IgG titers, with the 20 µg dose inducing titers comparable to the 5 µg dose. By contrast, no EV-A71-specific IgG was detected in the sera of mice vaccinated with empty LNPs. Notably, mice immunized with inactivated EV-A71 vaccine showed high antibody titers when ELISA plates were coated with inactivated EV-A71 virus particles (**Figure 2B**), while almost no antibodies were detected on plates coated with VP1 recombinant protein (**Figure 2C**). These data demonstrated that the EV-A71 mRNA vaccine could effectively elicit VP1 specific IgG antibodies.

To further investigate whether the sera from immunized mice have specific neutralizing antibody activity against EV-A71, micro-neutralization assays were conducted. As shown in **Figure 2D**, sera from the cohort immunized with either VP1 mRNA-LNP or the EV-A71 inactivated vaccine demonstrated strong neutralizing activity against EV-A71. Three weeks after the primary immunization, neutralizing antibodies titers reached 11.5 and 11.6 in the 5 μg and 20 μg mRNA vaccine groups, respectively. Following the second immunization, significantly increased neutralizing antibody titers were observed, reaching 21.2 in the 5 µg group and 54.4 in the 20 µg group. After the third immunization, titers further increased to 71.9 and 102.9, respectively. In contrast, all sera from mice immunized with empty LNPs showed no detectable neutralizing antibody titers, even at the lowest dilution (1:8). Nonetheless, the group immunized with the inactivated virus vaccine consistently displayed higher neutralizing antibody titers than the mRNA vaccine group, reaching 127.8 three weeks after the second booster immunization.

### Extraordinary cellular immune responses induced by EV-A71 VP1 mRNA-LNP in BALB/c mice

**Figure 3:**
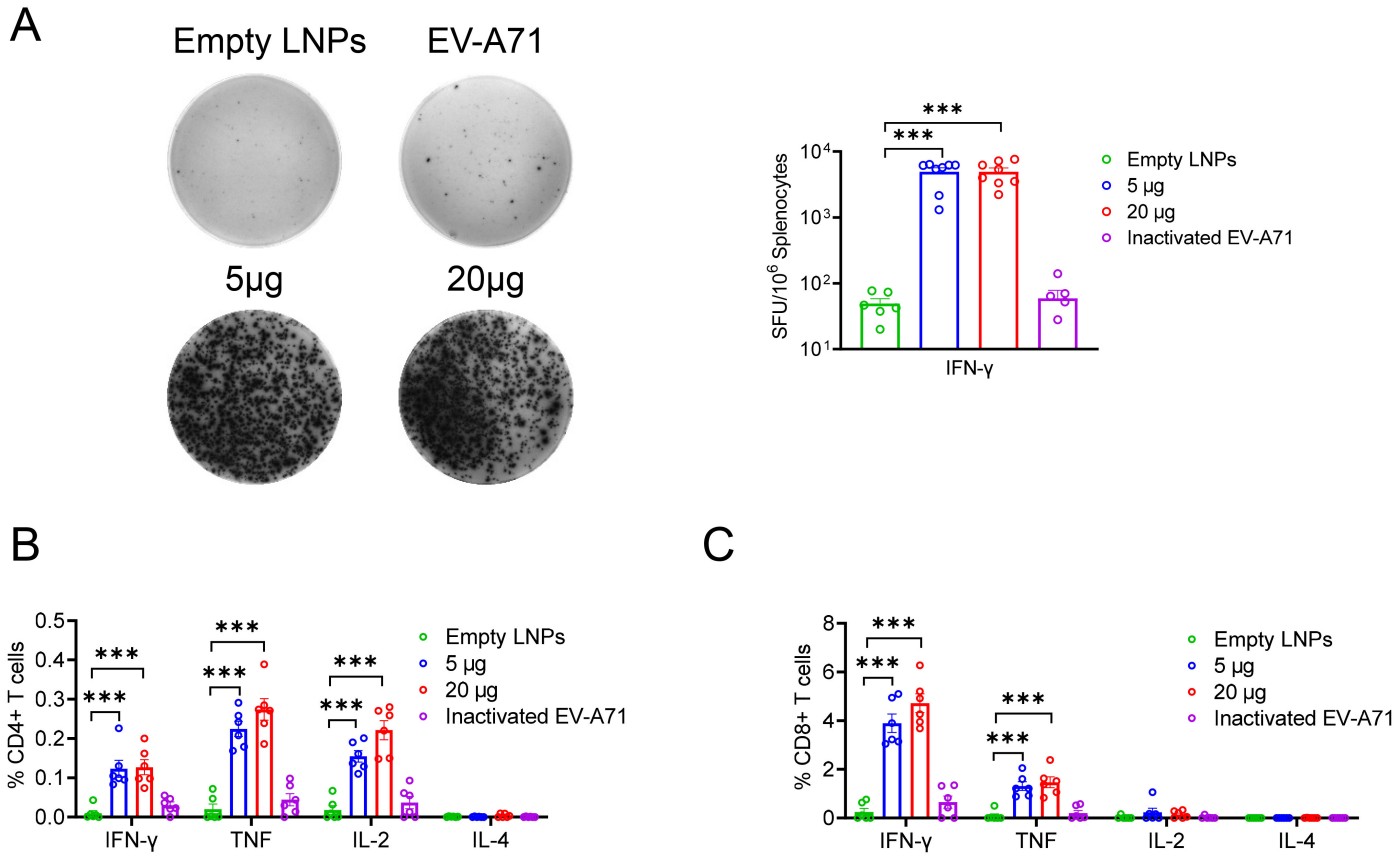
Cellular immune response induced by EV-A71 VP1 mRNA vaccines and inactivated EV-A71 vaccines in mice. **(A)** IFN-γ-producing T cells in spleen of mice immunized with vaccines were detected using the ELISPOT assay. The percentage of CD4^+^ **(B)** and CD8^+^ **(C)** T cells secreting IFN-γ, TNF, IL-2, or IL-4 were determined through intracellular cytokine staining (ICS). Data are shown as the mean ± SEM. Statistical significance was determined using unpaired Students’ t-test. ***p < 0.001.

**Figure 4:**
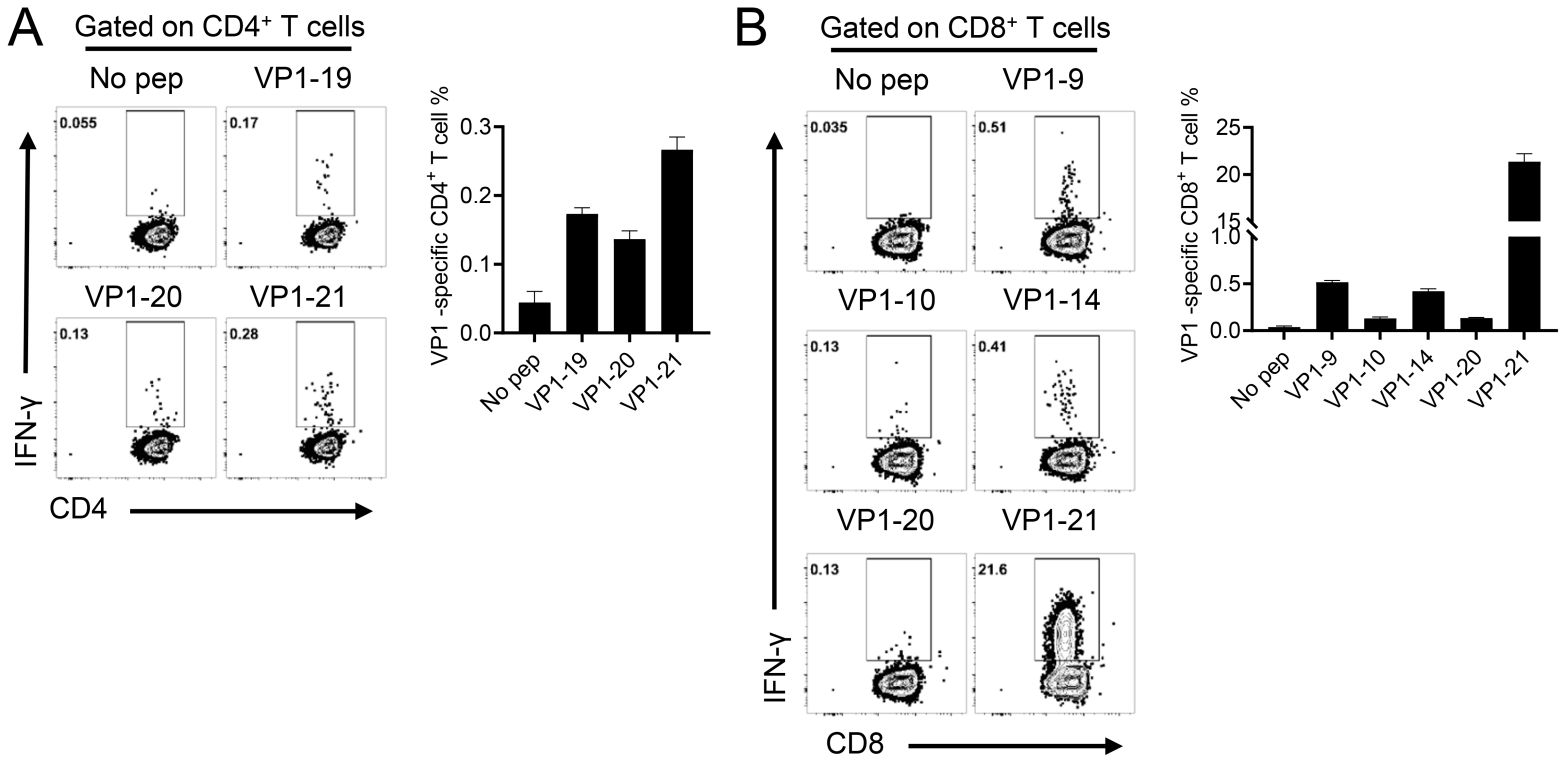
Identification of CD4^+^ and CD8^+^ T cell epitopes on EV-A71 VP1 mRNA in mice immunized with the EV-A71 VP1 mRNA vaccine. Confirmation of CD4 ^+^ **(A)** and CD8 ^+^ **(B)** T cell epitopes in infected BALB/c mice. Flow cytometry plots and summary data are presented. All results are expressed as mean ± SEM. pep, peptide.

To further assess whether cellular immune responses could be induced in mice by administering either EV-A71 VP1 mRNA-LNP or the EV-A71 inactivated vaccine, spleens were collected three weeks after the second booster immunization. The number of T cells secreting IFN-γ was measured using the enzyme-linked immunospot (ELISpot) assay. The results indicated that both the 5 μg and 20 μg doses of VP1 mRNA-LNP elicited a significantly higher number of IFN-γ-secreting cells (about 5000 spot-forming cells (SFC)/10^6^ cells) compared to mice immunized with empty LNPs or the inactivated EV-A71 vaccine, when stimulated with 10 μM peptide pool targeting EV-A71 VP1 (excluding peptide 7, which failed to be synthesized due to its high hydrophobicity) (**Figure 3A**). Furthermore, the specific T lymphocyte response was evaluated using an intracellular cytokine staining (ICS) assay. After stimulation with a 10 μM VP1 peptide pool for 5-6 h in the presence of brefeldin A, flow cytometry analysis was conducted. The result revealed that immunization with EV-A71 VP1 mRNA-LNP induced higher levels of VP1-specific CD4^+^ and CD8^+^ T cells secreting Th1-type cytokines, including IFN-γ, interleukin-2 (IL-2), and tumor necrosis factor (TNF), compared to those induced by empty LNPs. In contrast, the production of Th2-type cytokines such as IL-4 was extremely low in each immunization group, with no significant differences between groups (**Figures 3B, C**). Intriguingly, T cell responses in mice immunized with the inactivated EV-A71 vaccine were undetectable in either the ELISpot or ICS assay. These results suggested that immunization with the EV-A71 VP1 mRNA-LNP effectively elicited a robust Th1-biased specific T cellular immune response. Next, we investigate the T cell epitopes in BALB/c mice immunized with the VP1 mRNA-LNP vaccine, splenocytes were stimulated with 5 μM of each individual peptide for 5-6 h in the presence of brefeldin A, and VP1-specific T cells were identified by IFN-γ production using ICS. Three peptides (VP1-19, VP1-20, VP1-21) (**Figure 4A**) stimulated CD4^+^ T cells, while six peptides (VP1-9, VP1-10, VP1-14, VP1-20, VP1-21) stimulated CD8^+^ T cells (**Figure 4B**). Notably, peptides VP1-20 and VP1-21 exhibited dominant CD8 and CD4 T cell epitopes.

### Passive protection efficacy of mRNA vaccine against lethal viral challenge in neonatal mice

**Figure 5:**
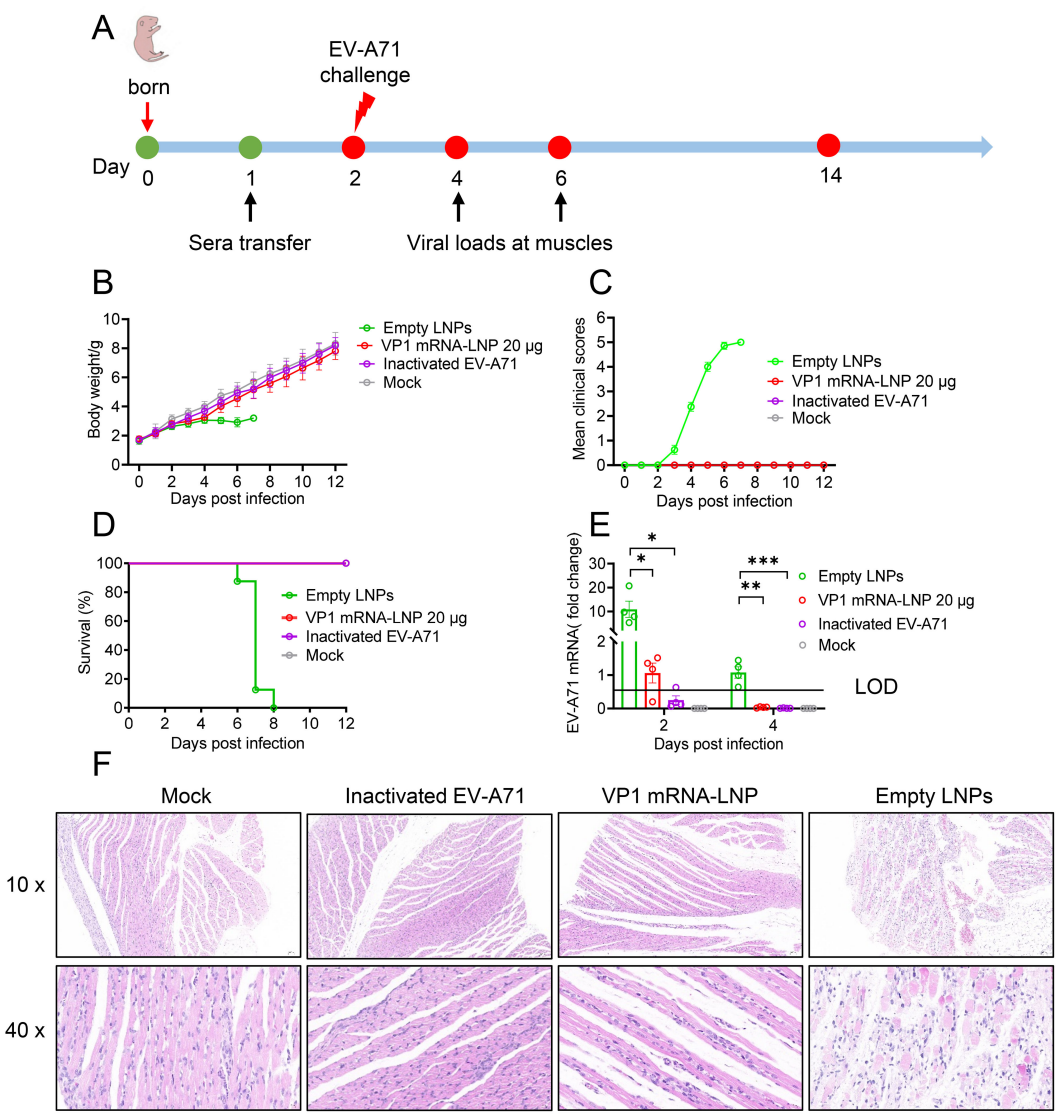
Protective efficacy of sera from vaccinated mice against lethal viral challenges in neonatal mice. **(A)** Schematic representation of the study design. One-day-old A129 mice were intraperitoneally injected (i.p.) with pooled sera from mice vaccinated with EV-A71 VP1 mRNA-LNP, inactivated EV-A71 or Empty LNPs. One day later, the mice were challenged with the EV-A71 virus and monitored daily for weight change **(B)**, clinical signs **(C)**, and survival **(D)**. **(E)** Two and four days after challenge, four mice from each group were selected, and their hind leg muscles were collected for viral RNA load detection. **(F)** Six days after challenge, two mice from each group were euthanized for pathological analysis of their hind leg muscles. Data are shown as the mean ± SEM. Statistical significance was determined using a two-way ANOVA with multiple comparison tests. *p < 0.05, **p < 0.01, ***p < 0.001.

To further evaluate *in vivo* protection efficacy of mRNA vaccine, one-day-old neonatal A129 mice received sera collected from mice three weeks after the second booster immunization intraperitoneally. Twenty-four hours later, the mice were challenged with a lethal dose of EV-A71 (2×10^7^ TCID_50_) (**Figure 5A**). The results showed that neonatal mice administrated sera from either VP1 mRNA-LNP-vaccinated or inactivated EV-A71-vaccinated mice exhibited a steady weight gain (**Figure 5B**), comparable to the mock group. No noticeable clinical symptoms were observed throughout the study period (**Figure 5C**), and all the mice survived (**Figure 5D**). In contrast, neonatal mice receiving sera from those vaccinated with empty LNPs showed significantly reduced weight gain since 5 dpi. Clinical symptoms began to manifest at 3 dpi and worsened over time, resulting in the death of all the mice by 8 dpi (**Figures 5B–D**).

Additionally, viral titers and pathological damage were evaluated in the hind leg muscles of neonatal mice at 2 and 4 dpi. Mice treated with sera from either the VP1 mRNA-LNP and inactivated EV-A71 vaccinated group showed significantly lower viral titers compared to the empty LNPs group (**Figure 5E**). In addition, histopathological analysis revealed severe muscle tissue damage in the mice treated with sera from empty LNPs immunized mice 6 dpi, characterized by extensive cell death, hemorrhage, moderate interstitial edema and disorganized connective tissue. In contrast, no such lesions were observed in the muscle sections of mice treated with sera from mRNA or inactivated-vaccinated mice or mock mice (**Figure 5F**). Overall, these findings demonstrate that mRNA vaccines provide complete protection against lethal EV-A71 infection in suckling mice model.

## Discussion

Since the late 1990s, EV-A71-induced hand, foot, and mouth disease (HFMD) has emerged as a significant public health challenge in the Asia-Pacific region. Based on the VP1 gene sequence, EV-A71 is currently classified into eight genotypes (A-H), with genotypes B and C being the most prevalent in this area. Genotype B is further divided into subtypes B0-B7, while genotype C includes subtypes C1-C6 (41). Notably, C4 sub-genotype is the dominant strain in Mainland China and has been responsible for several major outbreaks (9). Although three inactivated vaccines targeting the C4 sub-genotype have been authorized for use in Mainland China, new variants of EV-A71 are continuously emerging, and the production capacity for these vaccines cannot be rapidly scaled up. This situation poses significant challenges to achieving widespread immunization among high-risk populations during EV-A71 outbreaks (22). mRNA vaccines offer a potential solution due to their rapid development, scalable production and flexibility to quickly adapt formulations for emerging variants. Currently, mRNA vaccine research is primarily focused on enveloped viruses, but studies have demonstrated their effectiveness against non-enveloped viruses, such as enterovirus D68 (42).

Taking advantage of mRNA vaccine technology, we developed a novel vaccine targeting the prevalent EV-A71 C4 sub-genotype in this study. The immunogenicity and protective efficacy of the mRNA vaccine in murine models, comparing its performance with that of an inactivated EV-A71 vaccine. Our results indicated that the EV-A71 VP1 mRNA-LNP vaccine effectively induced VP1-specific IgG antibodies and neutralizing antibody responses, alongside a strong Th1-biased immune response. Notably, while the inactivated EV-A71 vaccines exhibit lower levels of total IgG antibodies on VP1-coated plates, and produced higher neutralizing antibody levels, which may contribute to the viral neutralizing epitopes present on the VP2 and VP3 proteins of the viruses. Previous researches have shown that neutralizing epitopes exist in the viral structural proteins VP2 (43, 44) and VP3 (13), and sera from immunized mice containing these neutralizing epitopes can protect against lethal challenges with EV-A71 *in vivo*. However, not all non-envelope proteins can be used to product effective mRNA vaccines with a single viral structural protein. For example, the individual expression of either the VP1 protein or P1 protein of EV-D68 does not elicit the production of neutralizing antibodies (42). Notablely, we detected relatively low levels of IgG titers of the inactivated vaccine immune sera on plates coated with the VP1 recombinant protein, which might be associated with the protein’s structure and the type of vaccine. Linear epitope recognition antibody detection in immune sera revealed significant differences between the two vaccine types; the mRNA vaccine elicited antibodies against the VP1 C-terminal sequence, whereas no significant linear epitope recognition antibodies against VP1 were detected in the inactivated vaccine immune sera (**Supplementary Figure S2**). This suggests that there are differences in the humoral immune responses induced by the two vaccines.

Additionally, the EV-A71 inactivated vaccine failed to elicit antigen-specific T cell immune responses, indicating that VP1 mRNA-LNP may provide more comprehensive immune protection. Previous studies have shown that severe cases of pulmonary edema induced by EV-A71 are associated with reduced level of Th1-biased cytokine, suggesting that the immunopathogenesis of EV-A71-related pulmonary edema may be linked to weakened cellular immunity (45). Recent studies on T-cell-mediated immunity against heterologous SARS-CoV-2 infection has demonstrated the importance of CD4^+^ and CD8^+^ T cells in providing protection, particularly through IFN-γ-mediated responses independent of antibodies (46). These findings underline the importance of Th1 cells in antiviral immunity, underscoring the need to evaluate vaccines based on their ability to elicit cellular immune responses in addition to antibody production. Nonetheless, it is important to acknowledge certain limitations in our study. Due to the lack of suitable animal models, we are currently unable to determine whether the T cell immune response induced by EV-A71 VP1 mRNA-LNP could effectively defend against lethal EV-A71 challenges.

Previous studies have shown that in neonatal mice, death is induced in an age- and dose-dependent manner, with a 100% mortality rate observed only in 1-day-old mice at higher inoculation doses (10^8^ PFU) (47). Additionally, neonatal mice typically exhibit a weak antibody response, and the production of neutralizing antibodies requires at least 14 days after immunization. By that time, these mice are no longer sensitive to enterovirus infection (48). Consequently, vaccine evaluations usually employ neonatal mouse models and vaccine efficacy is often determined through the passive transfer of immune sera or by immunizing the dams before gestation, allowing maternal antibodies to be conveyed to the suckling mice via the placenta (48–54). The EV-A71 isolate we used in this study was shown to be susceptible in IFNAR knockout neonatal mice rather than ICR mice. Therefore, A129 mice, deficiently in IFNα/β receptor, were subjected to EV-A71 infection for evaluation of our vaccines via adoptive transfer of sera. Both the EV-A71 VP1 mRNA-LNP and inactivated EV-A71 vaccines provided complete passive protection against lethal viral challenges, significantly reducing viral load and pathological lesions in both groups.

In conclusion, this study successfully developed an EV-A71 mRNA vaccine with strong immunogenicity and protective efficacy, making it a promising candidate for future vaccination strategies. We also identified several CD4^+^ and CD8^+^ T cell epitopes specific to the EV-A71 VP1 protein, which would serve as valuable targets for the future development of multi-epitope-based peptide vaccines.

## Materials and methods

### Virus, cells and mice

The EV-A71 C4 sub-genotype virus (JN315 strain, NMDC No. IMCN0006AUC) used for gene amplification, virus neutralization assays, and mouse infection experiments was isolated in our laboratory from clinical samples. The virus was propagated in RD cells, and the viral titer was determined. RD cells and Vero cells were maintained in Minimal Essential Medium (MEM) supplemented with 10% fetal bovine sera (FBS) at 37°C with 5% CO_2_, and HEK 293T and A549 cells were cultured in Dulbecco’s Modified Eagle Medium (DMEM) with 10% FBS under the same conditions. A129 mice, deficient in the interferon alpha/beta receptor were A129 mice were donated by the Institute Pasteur of Shanghai at the Chinese Academy of Sciences and housed at the Key Laboratory of Emerging Infectious Diseases in Universities of Shandong, Shandong First Medical University & Shandong Academy of Medical Sciences, BALB/c mice were purchased from Jinan Pengyue Experimental Animal Breeding Co., Ltd. All animal experiments were conducted in accordance with the People’s Republic of China legislation on the care and use of laboratory animals. The experimental protocols were approved by the Committee on the Ethics of Animal Experiments of Shandong First Medical University & Shandong Academy of Medical Sciences (Permit No. W202312230347).

### Design and production of nucleoside-modified VP1 mRNA-LNP

The VP1 structural protein sequence was derived from EV-A71 JN315 strain, with a secretion signal peptide (mouse Ig Kappa) added to the N-terminus. After codon optimization, the sequence was constructed into a eukaryotic expression plasmid (pCAGGS) and confirmed for expression in HEK 293T cells. Subsequently, 5’ and 3’ untranslated regions and a poly-A tail of Pfizer/BioNTech’s BNT162b2 mRNA (55) were added to both ends of the sequence. The sequence was then transcribed *in vitro* using T7 RNA polymerase to generate mRNA containing a 100 - nucleotides poly(A) tail, with uridine-5′-triphosphate substituted by m1Ψ-5′-triphosphate (TriLink BioTechnologies). After purification, the size and integrity of the mRNAs were analyzed by capillary electrophoresis. Lipid nanoparticles (LNPs) were encapsulated using a self-assembly process with the GenNano-E0011 reagent kit (Micro&Nano) and thoroughly mixed with the mRNA molecules using the INanoTML nanoparticle preparation system (Micro&Nano) to produce the VP1 mRNA-LNP vaccine. The average diameter of the VP1 mRNA-LNP was measured using Dynamic Light Scattering (DLS) with a Zetasizer Nano ZS (Malvern Instruments Ltd.).

### DNA and mRNA transfection

HEK 293T or RD cells were seeded in 24-well plates and incubated for 20 hours. The cells were then transfected with either pCAGGS-VP1 or VP1 transcribed mRNA using PEI Transfection Reagent (PEI-MAX 40K, 24765, Polysciences), taking the pCAGGS vector as a control. Additionally, VP1-mRNA-LNP was transfected following the same protocol. Four to six hours post transfection, the medium was replaced with DMEM containing 2% FBS. The cells were then processed for Western blot or immunofluorescence assays (IFA) to confirm protein production.

### Western blot

Cell lysate mixed with SDS-PAGE loading buffer (Solabio) was boiled and separated by SDS-polyacrylamide gel electrophoresis at 110 V for 1 hour, then transferred onto polyvinylidene fluoride (PVDF) membranes (Bio-Rad) at 240 mA for 2 hours. The membranes were blocked with 5% (w/v) skim milk for 2 hours at room temperature, followed by incubation with the primary (Rabbit anti-EV-A71 VP1 (GTX132338, GeneTex); Mouse anti-GAPDH (60004-1-Ig, Proteintech)), and secondary antibodies (Peroxidase AffiniPure donkey anti-mouse IgG (715-035-151, Jackson ImmunoResearch); Peroxidase AffiniPure donkey anti-rabbit IgG (711-035-152, Jackson ImmunoResearch)) after washing three times with 0.1% Tween Phosphate Buffered Saline (PBST) (Solabio). VP1 proteins were visualized using the Amersham Imager 600 ECL system (GE).

### Immunofluorescence assay

RD cells were transfected with EV-A71 VP1 mRNA molecules using PEI transfection reagent and incubated for 24 hours. The cells were then washed with PBS, fixed with 4% paraformaldehyde, and permeabilized with 0.2% Triton X-100 (X100, Sigma-Aldrich) at room temperature for 10 minutes. Following permeabilization, cells were stained with rabbit anti-VP1 primary antibody (GTX132338, GeneTex) for 2 hours at room temperature. Afterward, the cells were treated with Alexa Fluor 488 AffiniPure donkey anti-rabbit IgG (711-545-152, Jackson ImmunoResearch) and incubated with a nuclear staining dye DAPI (D9542, Sigma-Aldrich) in the dark. Finally, the samples were visualized using a ZEISS Observer 300 (Germany).

### Animal experiments

BALB/c mice were randomly assigned to four groups of six mice each. Two groups were vaccinated with either 5 μg or 20 μg of VP1 mRNA-LNP. The negative control group received empty LNPs, while the positive control group was immunized with a combination of inactivated EV-A71 virus combined with aluminum adjuvant. Immunizations were administered via the intramuscular (i.m.) route at three-week intervals. Blood samples were collected by submental bleeding on days 21, 42, and 63 after the primary vaccination to obtain sera for humoral immune response detection. On day 63, mice were anesthetized with 2,2,2-Tribromoethanol at a concentration of 20 μg/μL, with a dosage of 17.5 μL per gram of body weight. They were then euthanized by cervical dislocation, and their spleens were harvested and homogenized for T cell immune response analysis.

### Enzyme-linked immunosorbent assay

The total specific antibody titers in the sera were determined using ELISA. In brief, 96-well ELISA plates were coated with 200 μg/mL of either VP1 protein or inactivated EV-A71 virus and incubated overnight at 4°C. After four washes with PBST, plates were blocked with 100 μL of blocking buffer (1% BSA in PBST) for 2 hours at 37°C. Following three washes, 100 μL of serially diluted mouse sera were added to each well and incubated at 37°C for 2 hours. After four washes, 100 μL of HRP-conjugated goat anti-mouse IgG (1:10,000 dilution) (Solabio) was added and incubated for 1 hour at 37°C. The plates were washed four times and then developed with 50 μL/well of TMB solution (NCM Biotech) for 10-30 minutes at room temperature. The reaction was stopped by adding 50 μL/well of 2 mol/L sulfuric acid, and absorbance was measured at 450 nm (OD450).

### Virus neutralization assay

Vero cells were seeded at 10,000 cells per well in 96-well plates and maintained in DMEM supplemented with 10% FBS, then cultured for 20 hours at 37°C. Mouse sera were serially diluted starting from a 1:8 dilution in DMEM containing 2% FBS and incubated with equal volumes of a 100 TCID_50_ EV-A71 virus for 2 hours at 37°C. Then, 100 μL of the mixture was transferred to the Vero cells and incubated for another 2 hours. The cytopathic effect (CPE) in each well was assessed five days post-infection. Three replicates were conducted for each well, and 50% neutralizing antibody titer (NT_50_) were calculated. Negative control samples and virus back titrations were included to ensure the stability of the testing system.

### ELISpot assay

Cellular immune responses in vaccinated mice were evaluated using IFN-γ precoated ELISPOT kits (3321-4AST-2, MabTech) following the manufacturer’s protocol. The plates were analyzed with ImmunoSpot^®^ Analyzers to quantify the number of IFN-γ-secreting cells.

### Flow cytometry

The following monoclonal antibodies were used for surface and intracellular staining: anti-mouse CD4-A488 (100529, Biolegend), anti-mouse IL-2-PE (503808, Biolegend), anti-mouse CD16/CD32-PerCP-Cy5.5 (45-0161-82, eBioscience), anti-mouse TNF-APC-Cy7 (506344, Biolegend), anti-mouse CD8a-BV510 (100752, Biolegend), anti-mouse IL-4-PE-Cy7 (25-7041-80, eBioscience), anti-mouse IFN-γ-eFluor450 (48-7311-82, eBioscience). For intracellular cytokine staining (ICS), 1×10^6^ splenocytes were cultured in 96-well plates at 37°C for 5-6 hours in the presence of VP1 protein overlapping peptides and brefeldin A (BD Biosciences). The cells were then labeled for surface markers at 4°C for 15 minutes in the dark, followed by fixation and permeabilization using Cytofix/Cytoperm Solution (BD Biosciences) at 4°C for 30 minutes in the dark. Intracellular cytokines were stained using antibody cocktails. All flow cytometry data were collected on a BD FACSVerse Cytometer and analyzed using FlowJo software.

### Mouse challenge experiments

Passive immunization with antisera to assess protection against viral challenge in recipient mice was performed as follows: one-day-old A129 mice from five litters were mixed and randomly assigned into 4 groups (n=8), each group were injected intraperitoneally with 50 μL of pooled sera from the indicated vaccinated mice. Twenty-four hours later, the mice were challenged intramuscularly (i.m.) with a lethal dose of EV-A71 virus (2×10^7^ TCID_50/_50 μL). Weight change, clinical signs, and survival were monitored daily for up to 12 days post-infection (dpi). A repeat experiment was conducted, and four to five mice were euthanized on day 2 and 4 post-infection to evaluate virus titers in the hind leg muscles. On day 6 post-infection, two mice were euthanized to examine pathological changes in the hind leg muscles as previously described (56). All the suckling mice were euthanized by cervical dislocation. The clinical disease was scored as follows: 0, healthy; 1, lethargy and inactivity; 2, hind limb weakness; 3, single limb paralysis; 4, double hind limb paralysis; and 5, death (56).

### Statistical analysis

All statistical analysis was performed with GraphPad Prism version 5.0 (GraphPad 4 Software, San Diego, CA, USA). Analysis of variance (ANOVA) or t-test was used to determine statistical significance among different groups. Data were presented as means ± SEM. P values of <0.05 were considered statistically significant (n.s., not significant; *p < 0.05, **p < 0.01, ***p < 0.001, ****p < 0.0001).

## Data Availability

The datasets presented in this study can be found in online repositories. The names of the repository/repositories and accession number(s) can be found below: https://nmdc.cn/resource/genomics/sequence/detail/NMDCN0006AUC, IMCN0006AUC.
