# Supplementary material for: A nucleoside-modified mRNA vaccine prevents enterovirus A71 infection in mouse model
Source: Front Immunol. 2025 Feb 12;16:1535758. doi: 10.3389/fimmu.2025.1535758 (PMC11861539; doi:10.3389/fimmu.2025.1535758)
Supplement: Supplementary file 1 [file DataSheet1.docx]

Figure S1

| Anti-VP1 | 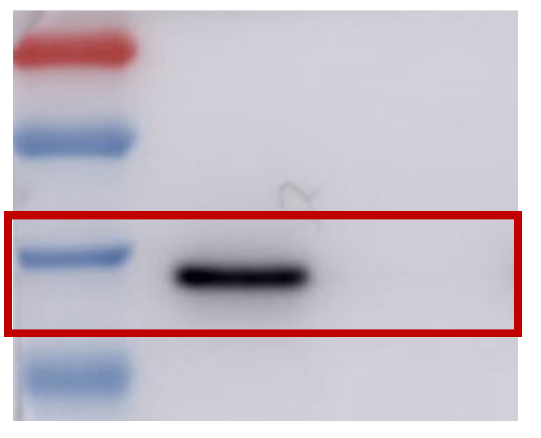 |
| --- | --- |
| Anti-GAPDH | 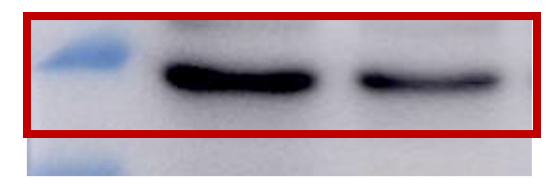 |
| Anti-VP1 | 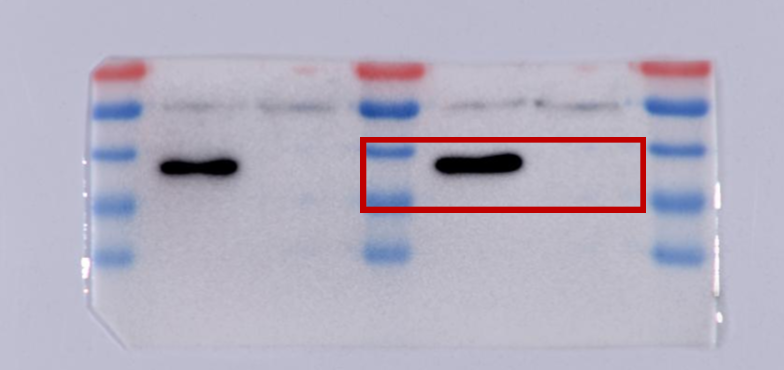 |
| Anti-GAPDH | 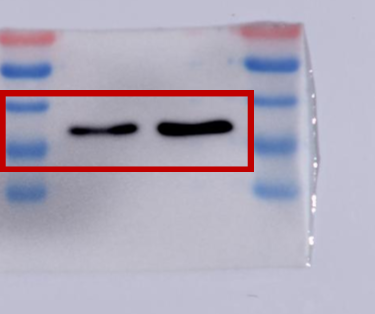 |
| Anti-VP1 | 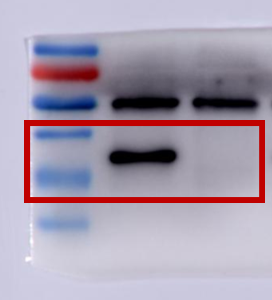 |
| Anti-GAPDH | 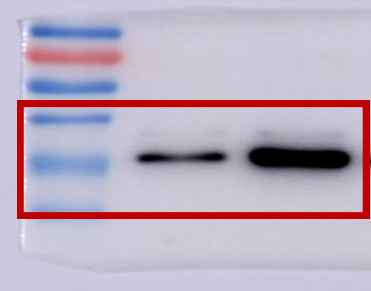 |

| 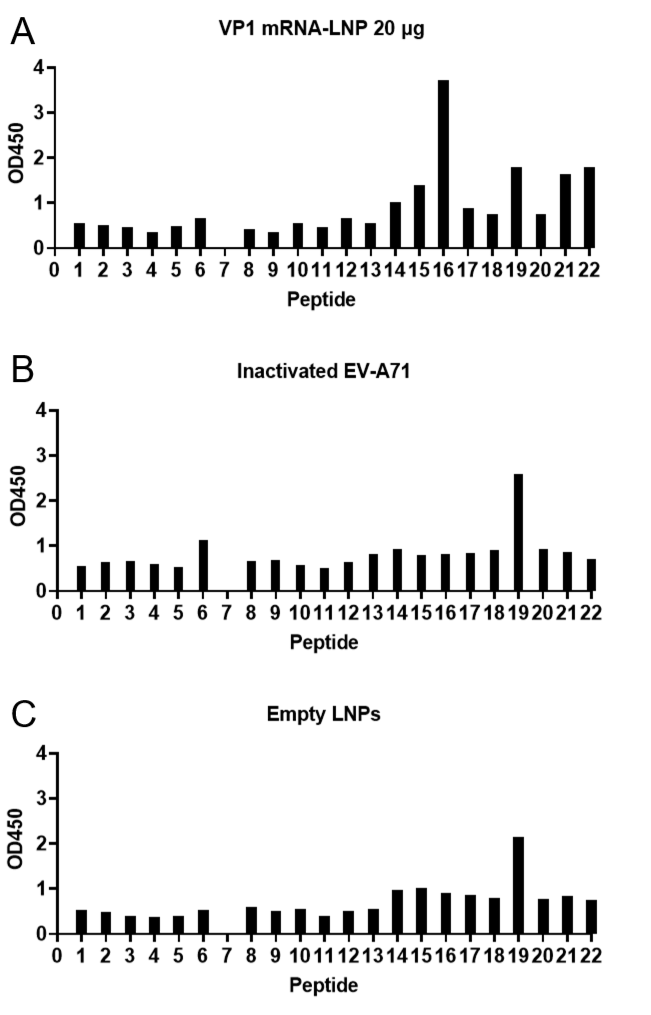 |
| --- |
| **Figure S2. Identification of EV-A71 VP1 B cell epitopes that elicit antibodies in the sera of vaccinated mice**. 96-well ELISA plates were coated with 10 ng/mL of each peptide and air-dried at room temperature, then peptide-specific linear antibodies in the mRNA vaccine (**A**) and inactivated (**B**) vaccine sera were determined using ELISA assay, Empty LNPs immunized mice sera used as control (**C**). |

| 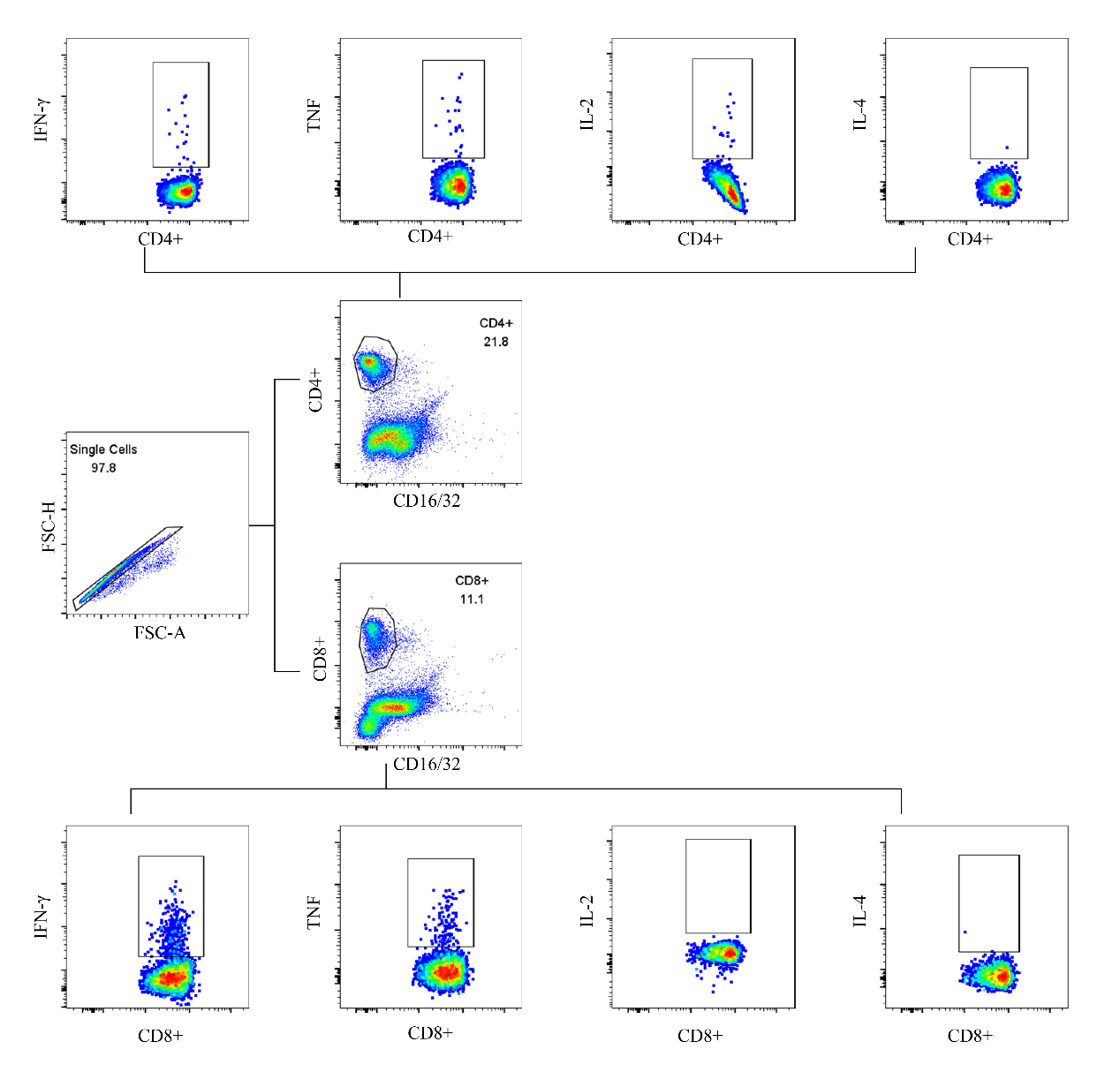 |
| --- |
| **Figure S3. Intracellular cytokine gating strategy for splenic EV-A71 VP1-specific CD4^+^ and CD8^+^ T cells** |

**Table S1. Amino acid sequence of the EV-A71 VP1 mRNA vaccine used in this study**

METDTLLLWVLLLWVPGSTGDGDRVADVIESSIGDSVSRALTHALPAPTGQNTQVSSHRLDTGKVPALQAAEIGASSNASDESMIETRCVLNSHSTAETTLDSFFSRAGLVGEIDLPLKGTTNPNGYANWDIDTTGYAQMRRKVELFTYMRFDAEFTFVACTPTGEVVPQLLQYMFVPPGAPKPDSRESLAWQTATNPSVFVKLSDPPAQVSVPFMSPASAYQWFYDGYPTFGEHKQEKDLEYGACPNNMMGTFSVRTVGTSKSKYPLVIRIYMRMKHVRAWIPRPMRNQNYLFKANPNYAGNSIKPTGASRTAITTL-

: Signal peptide

**Table S2. Overlapping peptides used in this study**

| Peptide No. | Sequence | Length (amino acids) |
| --- | --- | --- |
| Peptide-1 | ESMIETRCVLNSHSTAETTL | 20 |
| Peptide-2 | NSHSTAETTLDSFFSRAGLV | 20 |
| Peptide-3 | DSFFSRAGLVGEIDLPLKGT | 20 |
| Peptide-4 | GEIDLPLKGTTNPNGYANWD | 20 |
| Peptide-5 | TNPNGYANWDIDTTGYAQMR | 20 |
| Peptide-6 | IDTTGYAQMRRKVELFTYMR | 20 |
| Peptide-8 | FDAEFTFVACTPTGEVVPQL | 20 |
| Peptide-9 | TPTGEVVPQLLQYMFVPPGA | 20 |
| Peptide-10 | LQYMFVPPGAPKPDSRESLA | 20 |
| Peptide-11 | PKPDSRESLAWQTATNPSVF | 20 |
| Peptide-12 | WQTATNPSVFVKLSDPPAQV | 20 |
| Peptide-13 | VKLSDPPAQVSVPFMSPASA | 20 |
| Peptide-14 | SVPFMSPASAYQWFYDGYPT | 20 |
| Peptide-15 | YQWFYDGYPTFGEHKQEKDL | 20 |
| Peptide-16 | FGEHKQEKDLEYGACPNNMM | 20 |
| Peptide-17 | EYGACPNNMMGTFSVRTVGT | 20 |
| Peptide-18 | GTFSVRTVGTSKSKYPLVIR | 20 |
| Peptide-19 | SKSKYPLVIRIYMRMKHVRA | 20 |
| Peptide-20 | IYMRMKHVRAWIPRPMRNQN | 20 |
| Peptide-21 | WIPRPMRNQNYLFKANPNYA | 20 |
| Peptide-22 | YLFKANPNYAGNSIKPTGAS | 20 |
